# Bis(cyanato-κ*N*)bis­(5,7-dimethyl-1,2,4-triazolo[1,5-*a*]pyrimidine-κ*N* ^3^)zinc

**DOI:** 10.1107/S1600536811005769

**Published:** 2011-02-19

**Authors:** Ana B. Caballero, Miguel Quirós, Antonio Rodríguez-Diéguez, Juan M. Salas

**Affiliations:** a Departamento de Química Inorgánica, Facultad de Ciencias, Universidad de Granada, c/ Severo Ochoa s/n, 18071 Granada, Spain

## Abstract

In the title complex, [Zn(NCO)_2_(C_7_H_8_N_4_)_2_], the Zn^II^ ion exhibits a distorted tetra­hedral coordination geometry. The coordination environment is formed by two 5,7-dimethyl-1,2,4-triazolo[1,5-*a*]pyrimidine (dmtp) ligands, coordinated through the N atom in position 3, and two cyanate anions inter­acting by their N atoms. Supra­molecular dimers are generated by stacking inter­actions between the pyrimidine rings of two ligands related by an inversion center [centroid–centroid distance = 3.5444 (18) Å].

## Related literature

For similar structures, see: Adriaanse *et al.* (2009 ▶); Salas *et al.* (1999 ▶); Caballero *et al.* (2010 ▶). For a description of the geometry of tetra­hedrally coordinated metal atoms, see: Yang *et al.* (2007 ▶).
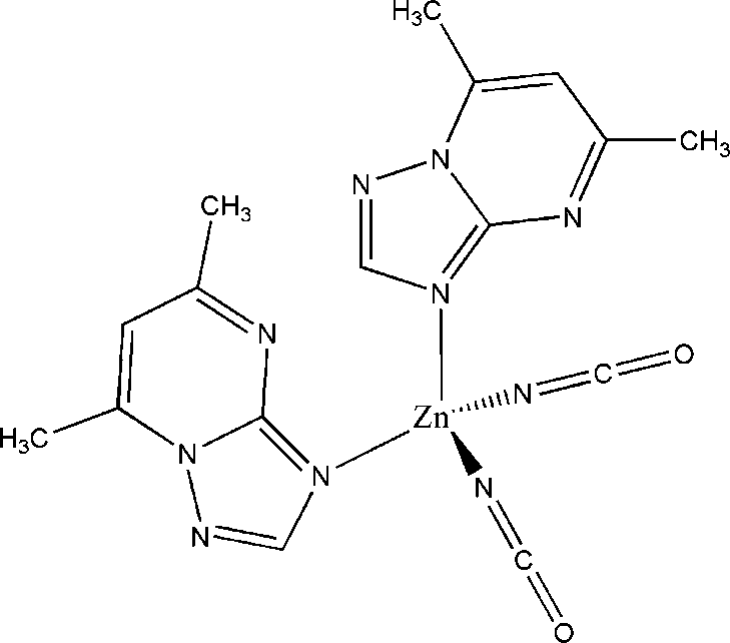


## Experimental

### 

#### Crystal data

[Zn(NCO)_2_(C_7_H_8_N_4_)_2_]
*M* *_r_* = 445.76
Triclinic, 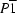

*a* = 10.0023 (15) Å
*b* = 10.8168 (16) Å
*c* = 11.1094 (16) Å
α = 116.772 (2)°
β = 107.226 (2)°
γ = 98.557 (2)°
*V* = 967.2 (2) Å^3^
*Z* = 2
Mo *K*α radiation
μ = 1.31 mm^−1^
*T* = 293 K
0.25 × 0.14 × 0.10 mm

#### Data collection

Bruker SMART APEX CCD system diffractometer
Absorption correction: multi-scan (*SADABS*; Sheldrick, 1996 ▶) *T*_min_ = 0.773, *T*_max_ = 0.881
11387 measured reflections
4403 independent reflections
3580 reflections with *I* > 2σ(*I*)
*R*_int_ = 0.025

#### Refinement

*R*[*F* ^2^ > 2σ(*F* ^2^)] = 0.039
*wR*(*F* ^2^) = 0.106
*S* = 1.01
4403 reflections
266 parameters
H-atom parameters constrained
Δρ_max_ = 0.37 e Å^−3^
Δρ_min_ = −0.22 e Å^−3^

### 

Data collection: *SMART* (Bruker, 2007 ▶); cell refinement: *SAINT* (Bruker, 2007 ▶); data reduction: *SAINT*; program(s) used to solve structure: *SHELXS97* (Sheldrick, 2008 ▶); program(s) used to refine structure: *SHELXL97* (Sheldrick, 2008 ▶); molecular graphics: Xtal_GX (Hall *et al.*, 1999 ▶); software used to prepare material for publication: *SHELXL97*.

## Supplementary Material

Crystal structure: contains datablocks global, I. DOI: 10.1107/S1600536811005769/su2253sup1.cif

Structure factors: contains datablocks I. DOI: 10.1107/S1600536811005769/su2253Isup2.hkl

Additional supplementary materials:  crystallographic information; 3D view; checkCIF report

## Figures and Tables

**Fig. 1.**
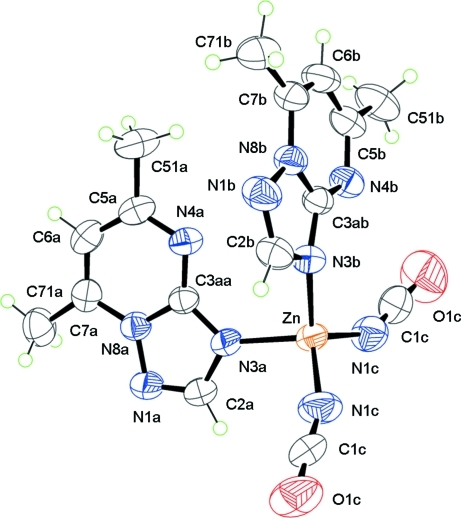
A view of the molecular structure of the title molecule with displacement ellipsoids drawn at the 50% probability level. H atoms are represented as spheres of arbitrary radii.

**Fig. 2.**
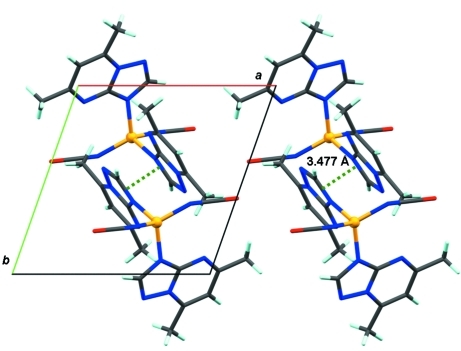
A view along c axis of the crystal packing of the title compound, showing the formation of the dimers by π···π interactions (dashed lines) involing pyrimidine ring (N4A, C3A, N8A, C7A, C6A, C5A) and that related by an inversion center.

## supplementary crystallographic information

### Comment

The coordination chemistry of 1,2,4-triazolo[1,5-*a*]pyrimidine derivatives displays great versatility, binding metal ions in several different ways, either in a monodentate (usually through the N atom in position 3) or in a bidentate fashion, bridging metal atoms and leading to dinuclear or polynuclear species with interesting metal-metal interactions (Salas *et al.*, 1999). Some zinc(II) complexes containing these derivatives together with secondary bridging ligands have been described, for example with the thiocyanate anion (Salas *et al.*, 1999; Adriaanse *et al.*, 2009). In most of these metal complexes, both ligands display monodentate binding leading to mononuclear species with either octahedral or tetrahedral coordination geometries.

The title compound continues our studies on a series of triazolopyrimidine and pseudohalide-based metal complexes (Caballero *et al.*, 2010). This zinc(II) complex, together with the analogous complex with the unsubstituted triazolopyrimidine ligand (Caballero *et al.*, 2010), are the only ones that have been obtained with the cyanate anion.

The title compound exhibits a distorted tetrahedral coordination geometry (τ_4_ = 0.924, Yang *et al.*, 2007) made of two dmtp ligands (dmtp = 5,7-dimethyl-1,2,4-triazolo[1,5-*a*]pyrimidine) interacting through their more usual coordination position, N3, and two cyanate anions bound through their N atom (Fig. 1). The Zn—N3 bond distances, 2.022 (2) and 2.043 (2) Å, are in the typical range for triazolopyrimidine ligands.

In the crystal the stacking interactions between the pyrimidine ring of two triazolopyrimidine aromatic systems leads to the formation of supramolecular centrosymmetric dimers (Fig. 2); the centroid-to-centroid distance, involving ring (N4A,C3A,N8A,C7A,C6A,C5A) and that related by an inversion center [symmetry code: 1-x, -y, -z], is 3.5444 (18) Å.

### Experimental

A 10 ml volume of an aqueous solution containing 1 mmol of NaNCO (0.068 g) was slowly added to a 10 ml aqueous solution containing 0.5 mmol of Zn(NO)_3_.4H_2_O (0.131 g) and 1 mmol of dmtp ligand (0.148 g). Immediately after adding NaNCO, a yellow turbidity gradually appeared. The mixture was stirred at 353 K for 15 min. and the precipitate was then filtered off. The resulting clear yellow solution was left to stand for a week at room temperature and yellow crystals of the title compound were collected and used for X-ray diffraction studies.

### Refinement

The pyrimidine H atoms were positioned geometrically and treated as riding with C—H = 0.93 Å (methine) and 0.96 Å (methyl), and with *U*_iso_(H) = 1.2*U*_eq_(C).

### Crystal data

**Table:**

[Zn(NCO)_2_(C_7_H_8_N_4_)_2_]	*Z* = 2
*M**_r_* = 445.76	*F*(000) = 456
Triclinic, *P*1	*D*_x_ = 1.531 Mg m^−^^3^
Hall symbol: -P 1	Mo *K*α radiation, λ = 0.71069 Å
*a* = 10.0023 (15) Å	Cell parameters from 3345 reflections
*b* = 10.8168 (16) Å	θ = 2.2–23.4°
*c* = 11.1094 (16) Å	µ = 1.31 mm^−^^1^
α = 116.772 (2)°	*T* = 293 K
β = 107.226 (2)°	Prismatic, colourless
γ = 98.557 (2)°	0.25 × 0.14 × 0.10 mm
*V* = 967.2 (2) Å^3^	

### Data collection

**Table:**

Bruker SMART APEX CCD system diffractometer	4403 independent reflections
Radiation source: fine-focus sealed tube	3580 reflections with *I* > 2σ(*I*)
graphite	*R*_int_ = 0.025
Detector resolution: 8.26 pixels mm^-1^	θ_max_ = 28.4°, θ_min_ = 2.2°
φ and ω scans	*h* = −13→12
Absorption correction: multi-scan (*SADABS*; Sheldrick, 1996)	*k* = −13→14
*T*_min_ = 0.773, *T*_max_ = 0.881	*l* = −14→14
11387 measured reflections	

### Refinement

**Table:**

Refinement on *F*^2^	Primary atom site location: heavy-atom method
Least-squares matrix: full	Secondary atom site location: difference Fourier map
*R*[*F*^2^ > 2σ(*F*^2^)] = 0.039	Hydrogen site location: inferred from neighbouring sites
*wR*(*F*^2^) = 0.106	H-atom parameters constrained
*S* = 1.01	*w* = 1/[σ^2^(*F*_o_^2^) + (0.060*P*)^2^ + 0.060*P*] where *P* = (*F*_o_^2^ + 2*F*_c_^2^)/3
4403 reflections	(Δ/σ)_max_ = 0.001
266 parameters	Δρ_max_ = 0.37 e Å^−^^3^
0 restraints	Δρ_min_ = −0.22 e Å^−^^3^

### Special details

**Table:**

Geometry. All e.s.d.'s (except the e.s.d. in the dihedral angle between two l.s. planes) are estimated using the full covariance matrix. The cell e.s.d.'s are taken into account individually in the estimation of e.s.d.'s in distances, angles and torsion angles; correlations between e.s.d.'s in cell parameters are only used when they are defined by crystal symmetry. An approximate (isotropic) treatment of cell e.s.d.'s is used for estimating e.s.d.'s involving l.s. planes.
Refinement. Refinement of *F*^2^ against ALL reflections. The weighted *R*-factor *wR* and goodness of fit *S* are based on *F*^2^, conventional *R*-factors *R* are based on *F*, with *F* set to zero for negative *F*^2^. The threshold expression of *F*^2^ > σ(*F*^2^) is used only for calculating *R*-factors(gt) *etc*. and is not relevant to the choice of reflections for refinement. *R*-factors based on *F*^2^ are statistically about twice as large as those based on *F*, and *R*- factors based on ALL data will be even larger.

### Fractional atomic coordinates and isotropic or equivalent isotropic displacement parameters (Å^2^)

**Table:**

	*x*	*y*	*z*	*U*_iso_*/*U*_eq_	
Zn	0.36171 (3)	0.27490 (3)	0.25442 (3)	0.04810 (12)	
N1A	0.7252 (2)	0.5596 (2)	0.2847 (2)	0.0566 (5)	
C2A	0.6371 (3)	0.5067 (3)	0.3287 (3)	0.0552 (6)	
H2A	0.6552	0.5497	0.4282	0.066*	
N3A	0.5182 (2)	0.3860 (2)	0.2224 (2)	0.0477 (4)	
C3AA	0.5321 (2)	0.3589 (2)	0.0970 (2)	0.0429 (5)	
N4A	0.4440 (2)	0.2520 (2)	−0.0413 (2)	0.0485 (5)	
C5A	0.4853 (3)	0.2523 (3)	−0.1441 (3)	0.0528 (6)	
C51A	0.3895 (4)	0.1323 (3)	−0.3011 (3)	0.0776 (9)	
H51A	0.4437	0.0690	−0.3384	0.093*	
H52A	0.3611	0.1745	−0.3605	0.093*	
H53A	0.3022	0.0766	−0.3053	0.093*	
C6A	0.6140 (3)	0.3580 (3)	−0.1100 (3)	0.0578 (6)	
H6A	0.6388	0.3529	−0.1861	0.069*	
C7A	0.7020 (3)	0.4665 (3)	0.0310 (3)	0.0514 (6)	
C71A	0.8386 (3)	0.5868 (3)	0.0821 (4)	0.0715 (8)	
H71A	0.8597	0.5736	−0.0010	0.086*	
H72A	0.9207	0.5851	0.1522	0.086*	
H73A	0.8231	0.6795	0.1281	0.086*	
N8A	0.6577 (2)	0.4640 (2)	0.1343 (2)	0.0455 (4)	
N1B	0.2927 (2)	−0.1274 (2)	−0.1076 (3)	0.0627 (6)	
C2B	0.3459 (3)	−0.0230 (3)	0.0314 (3)	0.0560 (6)	
H2B	0.4302	−0.0160	0.1018	0.067*	
N3B	0.2735 (2)	0.0740 (2)	0.0676 (2)	0.0463 (4)	
C3AB	0.1626 (2)	0.0281 (2)	−0.0619 (2)	0.0436 (5)	
N4B	0.0575 (2)	0.0863 (2)	−0.0882 (2)	0.0522 (5)	
C5B	−0.0380 (3)	0.0186 (3)	−0.2292 (3)	0.0580 (6)	
C51B	−0.1560 (3)	0.0829 (4)	−0.2625 (4)	0.0837 (10)	
H51B	−0.1447	0.1672	−0.1727	0.100*	
H52B	−0.2519	0.0113	−0.3059	0.100*	
H53B	−0.1470	0.1118	−0.3305	0.100*	
C6B	−0.0299 (3)	−0.1076 (3)	−0.3424 (3)	0.0645 (7)	
H6B	−0.0992	−0.1512	−0.4393	0.077*	
C7B	0.0770 (3)	−0.1662 (3)	−0.3123 (3)	0.0598 (7)	
C71B	0.0991 (4)	−0.2982 (3)	−0.4201 (3)	0.0881 (10)	
H71B	0.1921	−0.2690	−0.4245	0.106*	
H72B	0.0200	−0.3420	−0.5161	0.106*	
H73B	0.0995	−0.3682	−0.3894	0.106*	
N8B	0.1732 (2)	−0.0941 (2)	−0.1680 (2)	0.0484 (4)	
N1C	0.2232 (3)	0.3742 (3)	0.2904 (3)	0.0697 (6)	
C1C	0.1135 (4)	0.3882 (3)	0.2404 (4)	0.0699 (8)	
O1C	−0.0024 (3)	0.4026 (4)	0.1929 (4)	0.1284 (11)	
N1D	0.4708 (3)	0.2496 (3)	0.4110 (3)	0.0750 (7)	
C1D	0.5648 (3)	0.2391 (3)	0.4912 (3)	0.0589 (6)	
O1D	0.6598 (3)	0.2288 (3)	0.5767 (3)	0.0985 (8)	

### Atomic displacement parameters (Å^2^)

**Table:**

	*U*^11^	*U*^22^	*U*^33^	*U*^12^	*U*^13^	*U*^23^
Zn	0.05173 (19)	0.05143 (19)	0.03623 (16)	0.01493 (13)	0.01566 (13)	0.02134 (13)
N1A	0.0559 (12)	0.0492 (12)	0.0424 (11)	0.0070 (9)	0.0145 (10)	0.0137 (9)
C2A	0.0582 (15)	0.0539 (14)	0.0382 (12)	0.0137 (12)	0.0182 (11)	0.0150 (11)
N3A	0.0519 (11)	0.0467 (11)	0.0359 (10)	0.0134 (9)	0.0181 (9)	0.0159 (9)
C3AA	0.0468 (12)	0.0404 (11)	0.0395 (11)	0.0166 (10)	0.0168 (10)	0.0195 (10)
N4A	0.0568 (12)	0.0427 (10)	0.0376 (10)	0.0131 (9)	0.0154 (9)	0.0182 (9)
C5A	0.0708 (16)	0.0491 (14)	0.0399 (13)	0.0256 (12)	0.0209 (12)	0.0238 (11)
C51A	0.102 (2)	0.0684 (19)	0.0408 (15)	0.0189 (17)	0.0195 (15)	0.0212 (14)
C6A	0.0741 (17)	0.0644 (16)	0.0534 (15)	0.0314 (14)	0.0354 (14)	0.0368 (14)
C7A	0.0539 (14)	0.0562 (14)	0.0579 (15)	0.0245 (12)	0.0265 (12)	0.0362 (13)
C71A	0.0649 (18)	0.0762 (19)	0.083 (2)	0.0181 (15)	0.0344 (16)	0.0484 (18)
N8A	0.0488 (11)	0.0420 (10)	0.0424 (10)	0.0163 (9)	0.0177 (9)	0.0198 (9)
N1B	0.0521 (12)	0.0550 (13)	0.0588 (14)	0.0194 (10)	0.0156 (11)	0.0169 (11)
C2B	0.0480 (13)	0.0550 (15)	0.0550 (15)	0.0175 (11)	0.0122 (12)	0.0266 (13)
N3B	0.0467 (10)	0.0454 (10)	0.0398 (10)	0.0134 (8)	0.0117 (8)	0.0213 (9)
C3AB	0.0428 (12)	0.0406 (12)	0.0401 (12)	0.0076 (9)	0.0123 (9)	0.0204 (10)
N4B	0.0487 (11)	0.0485 (11)	0.0544 (12)	0.0142 (9)	0.0135 (10)	0.0285 (10)
C5B	0.0471 (14)	0.0557 (15)	0.0606 (16)	0.0056 (11)	0.0058 (12)	0.0359 (14)
C51B	0.0582 (18)	0.083 (2)	0.090 (2)	0.0150 (16)	−0.0003 (16)	0.0516 (19)
C6B	0.0555 (16)	0.0666 (17)	0.0458 (14)	−0.0007 (13)	0.0016 (12)	0.0281 (13)
C7B	0.0543 (15)	0.0522 (14)	0.0448 (14)	−0.0028 (12)	0.0137 (12)	0.0143 (12)
C71B	0.081 (2)	0.074 (2)	0.0547 (18)	0.0086 (17)	0.0222 (16)	0.0010 (15)
N8B	0.0459 (11)	0.0431 (10)	0.0429 (11)	0.0093 (8)	0.0131 (9)	0.0173 (9)
N1C	0.0721 (16)	0.0717 (15)	0.0620 (15)	0.0335 (13)	0.0318 (13)	0.0272 (13)
C1C	0.077 (2)	0.0734 (19)	0.073 (2)	0.0326 (17)	0.0411 (18)	0.0403 (17)
O1C	0.100 (2)	0.172 (3)	0.157 (3)	0.085 (2)	0.059 (2)	0.103 (3)
N1D	0.0783 (17)	0.0876 (18)	0.0526 (14)	0.0202 (14)	0.0111 (13)	0.0437 (14)
C1D	0.0783 (19)	0.0609 (16)	0.0416 (13)	0.0249 (14)	0.0291 (14)	0.0266 (12)
O1D	0.115 (2)	0.136 (2)	0.0756 (15)	0.0763 (18)	0.0359 (14)	0.0712 (16)

### Geometric parameters (Å, °)

**Table:**

Zn—N1C	1.902 (2)	N1B—C2B	1.306 (3)
Zn—N1D	1.919 (2)	N1B—N8B	1.370 (3)
Zn—N3B	2.0223 (19)	C2B—N3B	1.344 (3)
Zn—N3A	2.0430 (19)	C2B—H2B	0.9300
N1A—C2A	1.304 (3)	N3B—C3AB	1.339 (3)
N1A—N8A	1.372 (3)	C3AB—N4B	1.329 (3)
C2A—N3A	1.353 (3)	C3AB—N8B	1.365 (3)
C2A—H2A	0.9300	N4B—C5B	1.332 (3)
N3A—C3AA	1.344 (3)	C5B—C6B	1.414 (4)
C3AA—N4A	1.330 (3)	C5B—C51B	1.494 (4)
C3AA—N8A	1.373 (3)	C51B—H51B	0.9600
N4A—C5A	1.326 (3)	C51B—H52B	0.9600
C5A—C6A	1.411 (4)	C51B—H53B	0.9601
C5A—C51A	1.498 (4)	C6B—C7B	1.356 (4)
C51A—H51A	0.9603	C6B—H6B	0.9300
C51A—H52A	0.9604	C7B—N8B	1.357 (3)
C51A—H53A	0.9604	C7B—C71B	1.494 (4)
C6A—C7A	1.351 (4)	C71B—H71B	0.9602
C6A—H6A	0.9300	C71B—H72B	0.9602
C7A—N8A	1.357 (3)	C71B—H73B	0.9602
C7A—C71A	1.493 (4)	N1C—C1C	1.140 (4)
C71A—H71A	0.9601	C1C—O1C	1.188 (4)
C71A—H72A	0.9601	N1D—C1D	1.148 (3)
C71A—H73A	0.9601	C1D—O1D	1.188 (3)
			
N1C—Zn—N1D	115.09 (11)	N1A—N8A—C3AA	110.40 (18)
N1C—Zn—N3B	114.59 (9)	C2B—N1B—N8B	101.30 (19)
N1D—Zn—N3B	106.30 (9)	N1B—C2B—N3B	116.8 (2)
N1C—Zn—N3A	111.07 (9)	N1B—C2B—H2B	121.5
N1D—Zn—N3A	105.50 (10)	N3B—C2B—H2B	121.6
N3B—Zn—N3A	103.25 (8)	C3AB—N3B—C2B	103.40 (19)
C2A—N1A—N8A	101.70 (19)	C3AB—N3B—Zn	128.57 (15)
N1A—C2A—N3A	116.7 (2)	C2B—N3B—Zn	124.47 (16)
N1A—C2A—H2A	121.6	N4B—C3AB—N3B	128.1 (2)
N3A—C2A—H2A	121.6	N4B—C3AB—N8B	124.0 (2)
C3AA—N3A—C2A	103.34 (19)	N3B—C3AB—N8B	107.9 (2)
C3AA—N3A—Zn	130.18 (16)	C3AB—N4B—C5B	115.2 (2)
C2A—N3A—Zn	126.47 (16)	N4B—C5B—C6B	122.5 (2)
N4A—C3AA—N3A	128.6 (2)	N4B—C5B—C51B	116.4 (3)
N4A—C3AA—N8A	123.6 (2)	C6B—C5B—C51B	121.1 (3)
N3A—C3AA—N8A	107.82 (19)	C5B—C51B—H51B	109.6
C5A—N4A—C3AA	115.4 (2)	C5B—C51B—H52B	109.7
N4A—C5A—C6A	122.6 (2)	H51B—C51B—H52B	109.5
N4A—C5A—C51A	116.8 (2)	C5B—C51B—H53B	109.2
C6A—C5A—C51A	120.5 (2)	H51B—C51B—H53B	109.5
C5A—C51A—H51A	109.6	H52B—C51B—H53B	109.5
C5A—C51A—H52A	109.5	C7B—C6B—C5B	121.2 (2)
H51A—C51A—H52A	109.4	C7B—C6B—H6B	119.4
C5A—C51A—H53A	109.5	C5B—C6B—H6B	119.4
H51A—C51A—H53A	109.4	C6B—C7B—N8B	114.9 (2)
H52A—C51A—H53A	109.4	C6B—C7B—C71B	127.0 (3)
C7A—C6A—C5A	121.3 (2)	N8B—C7B—C71B	118.1 (3)
C7A—C6A—H6A	119.3	C7B—C71B—H71B	109.2
C5A—C6A—H6A	119.3	C7B—C71B—H72B	109.3
C6A—C7A—N8A	115.0 (2)	H71B—C71B—H72B	109.5
C6A—C7A—C71A	126.8 (2)	C7B—C71B—H73B	109.9
N8A—C7A—C71A	118.1 (2)	H71B—C71B—H73B	109.5
C7A—C71A—H71A	109.7	H72B—C71B—H73B	109.4
C7A—C71A—H72A	109.5	C7B—N8B—C3AB	122.2 (2)
H71A—C71A—H72A	109.5	C7B—N8B—N1B	127.2 (2)
C7A—C71A—H73A	109.3	C3AB—N8B—N1B	110.57 (19)
H71A—C71A—H73A	109.5	C1C—N1C—Zn	146.6 (2)
H72A—C71A—H73A	109.5	N1C—C1C—O1C	177.2 (4)
C7A—N8A—N1A	127.6 (2)	C1D—N1D—Zn	161.5 (3)
C7A—N8A—C3AA	122.0 (2)	N1D—C1D—O1D	178.1 (3)
